# A184 REPURPOSING DRUGS FOR SPLEEN TYROSINE KINASE (SYK) PEDIATRIC PATIENTS USING HIGH-THROUGHPUT SCREENING

**DOI:** 10.1093/jcag/gwab049.183

**Published:** 2022-02-21

**Authors:** S Chau, S Jardine, C Guo, N Warner, A Muise

**Affiliations:** 1 University of Toronto, Toronto, ON, Canada; 2 The Hospital for Sick Children, Toronto, ON, Canada

## Abstract

**Background:**

Spleen Tyrosine Kinase (SYK) is a cytosolic, non-receptor tyrosine kinase with an imperative role in immune and non-immune processes. Recently, we identified six gain-of-function SYK variants in patients that presented multi-organ inflammation and immune dysregulation. The SYK variants displayed constitutive SYK phosphorylation in human embryonic kidney (HEK) 293T, colonic epithelial cells (SW480), and in knock-in heterozygous SYK mice. These observations mark SYK as a therapeutic target for autoimmune diseases.

Phenotype drug discovery accelerates this process and can be done successfully with an appropriate phenotype. A possible phenotype displayed by SYK variants is SYK phosphorylation, as high-throughput screening can identify hit compounds that reduce the constitutive activation of phosphorylated SYK (p-SYK).

**Aims:**

**Aim 1: Determine the screening phenotype with wildtype (WT) and *SYK* S550Y variant in HEK293T cells.** Recently, we observed increased phosphorylation in gain-of-function *SYK* variants We hypothesize that we can use phosphorylated-SYK (p-SYK) levels to identify hit compounds that can decrease the kinase activity in these variants. With stable transfected SYK WT and SYK S550Y HEK293T cell-line, protein analyses will be completed to characterize the appropriate screening phenotype.

**Aim 2:**

**Establish an assay for high-throughput drug screening.** We will utilize homogenous time-resolved fluorescence (HTRF) assay. The signal measured from HTRF is positively proportional to the level of p-SYK; therefore, we expect that S550Y cells will have a higher signal than the WT.

**Aim 3:**

**Validate hit compounds in HEK293T and zebrafish.** We will create a dose-response curve with the hit compounds in *in vitro* and *in vivo* models.

**Methods:**

We will use stable transfection to established overexpressing SYK WT and S550Y HEK293T cells. We will apply homogenous time-resolved fluorescence (HTRF) to quantify p-SYK levels during the drug screening.

**Results:**

Protein analyses have verified high expression of p-SYK in stable transfected HEK293T cells. No stimulation was required, as the cells showed an increased phosphorylation level at baseline. Downstream signaling partners such as p-ERK and p-JNK of the MAPK pathway displayed an upregulation. This suggests that the sustained activation of p-SYK may consequently affect cellular processes and contribute to the clinical manifestations observed in patients.

**Conclusions:**

This research study will identify hit compounds that can produce a safe and effective biological response in pediatric patients with gain-of-function SYK variants. Personalizing medicine throughout high-throughput drug screening can accelerate drug repurposing for pediatric patients with multiple systemic diseases and immune dysregulation.

**Funding Agencies:**

None

